# Mechanism and Progress of Natural Products in the Treatment of NAFLD-Related Fibrosis

**DOI:** 10.3390/molecules28237936

**Published:** 2023-12-04

**Authors:** Jin-Zhong Li, Ning Chen, Nan Ma, Min-Ran Li

**Affiliations:** 1 Division of Infectious Disease, The First Affiliated Hospital, Jinan University, Guangzhou 510632, China; 2 General Medicine, The First Affiliated Hospital, Jinan University, Guangzhou 510632, China; 3 Center for Bioactive Natural Molecules and Innovative Drugs Research, College of Pharmacy, Jinan University, Guangzhou 510632, China; 4 JNU-HKUST Joint Laboratory for Neuroscience and Innovative Drug Research, College of Pharmacy, Jinan University, Guangzhou 510632, China

**Keywords:** NAFLD, NASH, liver fibrosis, natural products

## Abstract

Nonalcoholic fatty liver disease (NAFLD) has emerged as the most prevalent chronic liver disorder worldwide, with liver fibrosis (LF) serving as a pivotal juncture in NAFLD progression. Natural products have demonstrated substantial antifibrotic properties, ushering in novel avenues for NAFLD treatment. This study provides a comprehensive review of the potential of natural products as antifibrotic agents, including flavonoids, polyphenol compounds, and terpenoids, with specific emphasis on the role of Baicalin in NAFLD-associated fibrosis. Mechanistically, these natural products have exhibited the capacity to target a multitude of signaling pathways, including Hedgehog, Wnt/β-catenin, TGF-β1, and NF-κB. Moreover, they can augment the activities of antioxidant enzymes, inhibit pro-fibrotic factors, and diminish fibrosis markers. In conclusion, this review underscores the considerable potential of natural products in addressing NAFLD-related liver fibrosis through multifaceted mechanisms. Nonetheless, it underscores the imperative need for further clinical investigation to authenticate their effectiveness, offering invaluable insights for future therapeutic advancements in this domain.

## 1. Introduction

Nonalcoholic fatty liver disease (NAFLD) has become the leading chronic liver disease worldwide and is characterized by the accumulation of more than 5% of fat in liver cells [1]. Nonalcoholic steatohepatitis (NASH) is characterized by inflammation, hepatocyte ballooning, and necrosis and gradually progresses to fibrosis, which is dominated by hepatic stellate cells (HSCs) and the excess accumulation of extracellular matrix (ECM) proteins [2]. In large-scale biopsy-confirmed NAFLD studies, the presence of NASH does not increase the risk of liver-specific incidence or overall mortality. However, the risk of liver-related mortality increases exponentially with the progression of fibrosis, suggesting that liver fibrosis (LF) is the only independent correlate of total mortality in NAFLD patients [3,4].

## 2. Mechanisms of LF in Fatty Liver Disease

Liver tissue repair involves the concerted actions of various cell types (HSCs, hepatocytes, liver progenitor cells, endothelial cells, and immune cells) [5]. Persistent activation of HSCs and abnormal reprogramming of liver progenitor cells lead to excessive collagen deposition and accumulation during chronic liver injury [6]. The result of fibrosis is the continued and even amplified production of fibrotic cells through preferential recruitment rather than the decomposition of the fibrotic subpopulation [7].

Activated HSCs are the major precursors of activated myofibroblasts, which are the primary source of ECM. Transforming growth factor-beta (TGF-β) and platelet-derived growth factor are two major cytokines that promote the activation and proliferation of HSCs [8]. The activation of myofibroblasts occurs through a common mechanism called epithelial–mesenchymal transition (EMT), wherein quiescent hepatic stellate cells (Q-HSCs) with an epithelial phenotype, liver progenitor cells, bile duct epithelial cells, and sinusoidal endothelial cells transform into mature myofibroblasts with a mesenchymal phenotype [9]. Many other cellular factors, intracellular signaling pathways, and transcription factors are involved in this process. Thus, inhibiting HSC activation is a key factor in preventing the development of LF. HSCs participate in the development of LF through multiple signaling pathways, including TGF-β/Smad, PI3K/Akt, Notch, RAS/ERK, Wnt, Hedgehog, and P38MAPA [10]. Different mechanisms and the integration of multiple signals from hepatocytes, immune cells, and extracellular tissues generate a coherent reparative response.

### 2.1. Hedgehog (Hh) Signaling

**Figure 1:**
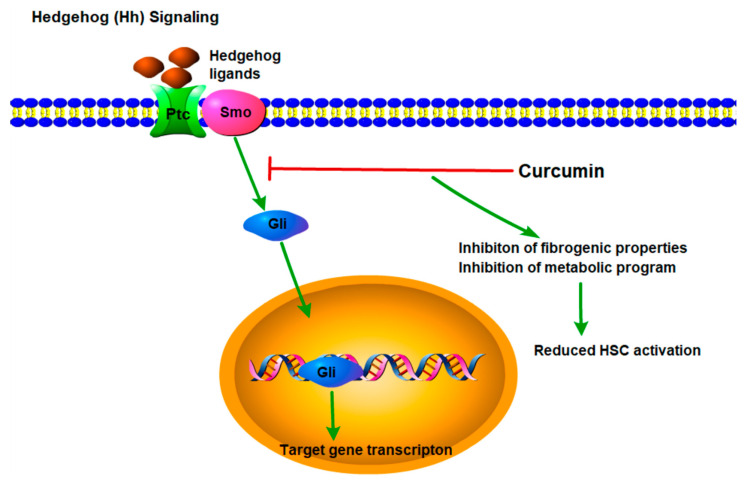
Hedgehog (Hh) signaling pathway and target of natural compounds.

Hedgehog (Hh) is a classic morphogen secreted by ligand-producing cells that diffuses into the extracellular space to regulate Hh-responsive target cells [11]. Hh regulates various biological processes, including proliferation, differentiation, vitality and adult liver regeneration, in Hh receptor-expressing cells [12]. Hh activation stimulates Hh-responsive cells to produce other factors that regulate injury repair. For example, Hh signaling induces HSCs to express TGF-β, CTGF, amphiregulin, jagged and Wnt ligands [13]. Hh stimulates the production of vascular endothelial growth factor by hepatic sinusoidal endothelial cells and induces the expression of osteopontin and chemokines by catheter cells, which recruit various types of immune cells to the damaged liver [14]. These immune cells further secrete multiple cytokines, such as interleukin (IL)-1β, IL-6, and tumor necrosis factor-alpha (TNF-α), exacerbating liver inflammation and damage. Additionally, Hh regulates macrophage polarization, thereby modulating the local balance of inflammatory, anti-inflammatory and fibrotic cytokines [15]. Studies have shown a relationship between Hh signaling pathway activation and liver progenitor cell generation in NAFLD, and the Hh pathway promotes liver progenitor cell proliferation to replace damaged hepatocytes with newly regenerated healthy hepatocytes [16,17]. While Hh signaling is necessary for injured adult livers to regenerate, chronic inflammation and fibrosis are caused when the pathway activation is excessive and/or prolonged [16]. Research has demonstrated that Q-HSCs express high levels of the Hedgehog-interaction protein (Hhip) [18]. After 24 h of culture in a serum-containing matrix, the expression of Hhip decreased by 90%, which was accompanied by the production of the sonic hedgehog (Shh) ligand and activation of the Hh signaling pathway. Activation of the Hh pathway also occurs in the methionine–choline deficient (MCD) diet-induced model of NASH fibrosis, and transgenic mice exhibit greater Hh pathway activation than wild-type mice, resulting in more severe fibrosis [19]. Inhibiting the Hh pathway via drug intervention (such as cyclopamine or GDC-0449, which are both Smo antagonists) can prevent the progression of LF (Figure 1) [12,20].

### 2.2. TGF-β1

**Figure 2:**
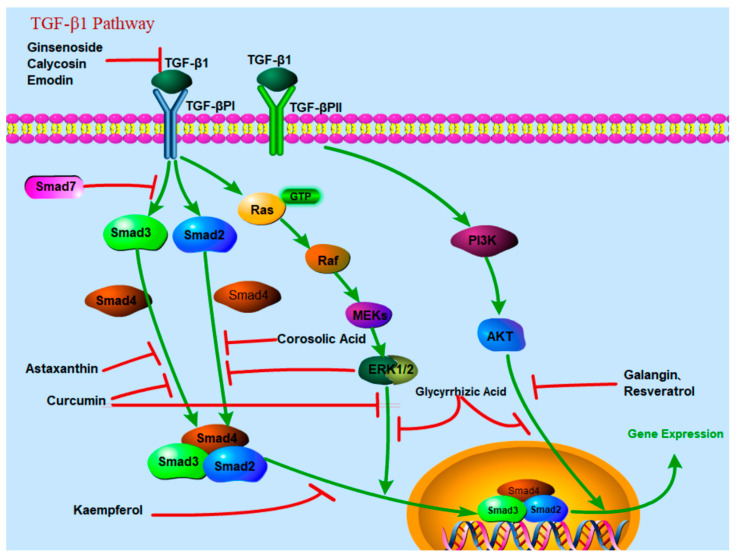
TGF-β1 signaling pathway and target of natural compounds.

TGF-β is the strongest profibrotic cytokine that is upregulated during LF and a strong inducer of EMT [21,22]. This factor can induce fibrosis by activating HSCs and liver progenitor cells, stimulating ECM synthesis, and inhibiting matrix degradation through the production of tissue inhibitors of metalloproteinases (TIMP)-1 [23]. The TGF-β family contains five subtypes. Generally, TGF-β1 is the most widely and deeply studied subtype in LF [24,25]. Shortly after liver injury, liver parenchymal cells and activated HSCs produce a large amount of TGF-β1. When TGF-β1 binds to the TGF-β Ⅱ receptor in the cell membrane of HSCs, the Ⅱ receptor phosphorylates the Ⅰ receptor, and the activated Ⅰ receptor induces the phosphorylation of Smad2 and Smad3. After being phosphorylated, Smad2, Smad3 and Smad4 form a complex, which is transferred into the nucleus. Smad3 binds to the promoter region of collagen, stimulating its transcription and producing a large amount of ECM [26]. However, TGF-β1 also plays an important physiological role in many aspects of cell proliferation, development, apoptosis and other biological processes. It is not feasible to widely target TGF-β1 as an antifibrotic strategy because of its functional diversity and pleiotropic effects. In a recent study, the inhibition of TGF-β type I receptor (ALK5) was coupled to mannose 6-phosphate human serum albumin (M6PHSA), and M6PHSA specifically delivered the ALK5 inhibitor to HSCs [27]. And, HSC αv integrin depletion inhibits fibrosis by reducing TGF-β activation (Figure 2) [28].

### 2.3. Wnt/β-Catenin

**Figure 3:**
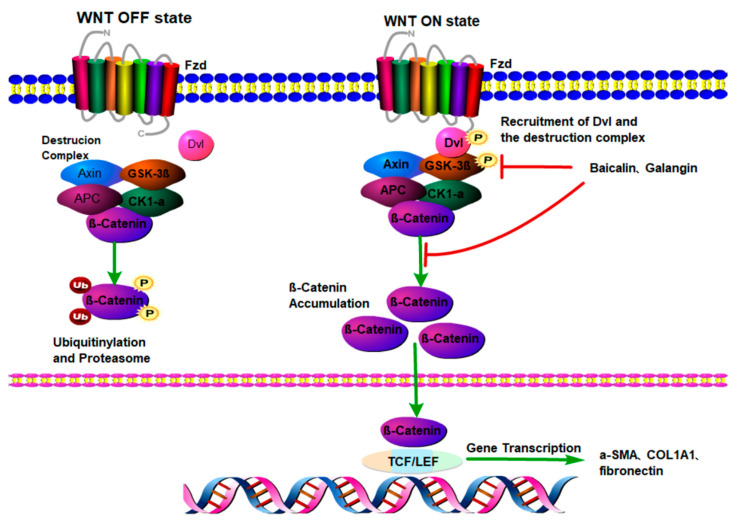
Wnt/β-catenin signaling pathway and target of natural compounds.

The Wnt/β-catenin signaling pathway is responsible for normal development, regeneration, metabolic partitioning and hepatobiliary development in the liver and maintains liver homeostasis [29]. The results show that the Wnt signaling pathway is closely related to the activation and proliferation of HSCs and LF [30]. Canonical Wnt signaling depends on β-catenin. The binding of Wnt ligands induces spatial interactions between cell surface receptors and their coreceptors, forming ternary complexes [31]. Ligand binding causes receptor conformational changes and then activates the downstream Wnt signaling pathway. After being activated, β-catenin is transferred to the nucleus, triggering the expression of Wnt target genes (Figure 3) [32]. A study involving rat HSC lines proved that β-catenin was highly expressed in the nucleus of activated HSCs, and siRNA-mediated knockout of β-catenin could inhibit HSC proliferation, increase apoptosis and inhibit the synthesis of type I and type III collagen [33], which indicated that β-catenin siRNA alleviated LF by controlling the activation of HSCs [34].

## 3. Natural Products with Potential Activity

At present, nearly half of the drugs used in liver treatment are natural products or derivatives of natural products [35,36,37]. Many natural products, which are mainly derived from plants, contain many active ingredients [38,39]. Because of their relative applicability, effectiveness and safety, natural drugs are now growing globally [40]. Recent investigations on functional foods show that many natural preparations have protective and therapeutic effects on the liver. Herbs and nutritional supplements also make them beneficial to the liver [41]. The natural product exerts anti-fibrosis effects by blocking signaling pathways such as Hedgehog, Wnt/β-catenin, TGF-β1, and NF-κB (Table 1).

### 3.1. Flavonoids

Flavonoids are polyphenols with a C3-C6-C3 core structure. Because phenolic hydroxyl groups are connected to different functional groups, they exhibit different biological activities. Most flavonoids protect the liver, inhibit oxidation, inflammation, diabetes, and cardiovascular disease and have immunomodulatory effects [83,84].

#### 3.1.1. Baicalin

**Figure 4:**
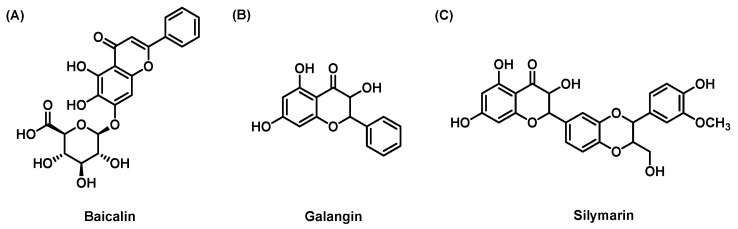
The chemical structures of (**A**) Baicalin; (**B**) Galangin; (**C**) Silymarin.

Baicalin (Figure 4A) is a kind of flavonoid compound extracted from the dried root of *Scutellaria baicalensis*. A large number of in vitro and in vivo studies show that baicalin has different pharmacological properties, including antioxidant, anti-inflammatory and hepatoprotective properties. These biological properties can be attributed to the fact that baicalin can target multiple pathways and bind to multiple signaling molecules [85]. A mouse model of NAFLD induced by an MCD diet showed that baicalin treatment significantly inhibited liver inflammation induced by MCD. This outcome was also related to decreases in serum TNF-α, IL-1β and monocyte chemoattractant protein-1 (MCP-1) levels, the inhibition of macrophage influx, and the activation of nuclear factor kappa-light-chain-enhancer of activated B cells (NF-κB). In addition, baicalin inhibits hepatic fibrosis by inhibiting α-smooth muscle actin (α-SMA), TGF-β1 and COL1A1 production [42]. A recent study showed that baicalin (200 mg/kg) could reduce the expression of fibrosis-related genes such as α-SMA, connective tissue growth factor and inflammatory factors such as TNF-α, macrophage inflammatory protein-1α, IL-1β and macrophage inflammatory protein-2, thus effectively inhibiting LF. In vitro studies also showed that baicalin could inhibit the activation of HSCs and downregulate the expression of α-SMA, fibronectin, TIMP1 and collagen 1 [43].

Many mechanisms of these therapeutic effects have been revealed. For example, baicalin decreased the expression of miR-3595, increased the activity of long-chain fatty acid coenzyme A ligase 4, and significantly inhibited the activity of HSCs, resulting in a decrease in fibrosis in HSC-T6 hepatocytes caused by platelet-derived growth factor [86]. In addition, baicalin inhibits PPAR-γ through Wnt signaling, which can reduce the activity of HSCs [87,88]. Baicalin alleviates LF induced by carbon tetrachloride (CCl4) in mice by regulating TGF-β1, hydroxyproline, procollagen type III, laminin (LN) and hyaluronic acid (HA). Baicalin can also reduce LF by inhibiting the activities of superoxide dismutase (SOD) and glutathione peroxidase (GPx) [44].

#### 3.1.2. Galangin

Galangin (GA, Figure 4B) (3,5,7-trihydroxyflavone) is a natural polyphenol compound extracted from the rhizome of *Alpinia officinarum*. Studies have reported various pharmacological properties of GA, such as the inhibition of inflammation, oxidation, tumors, allergy and Alzheimer’s disease [89,90]. LX-2 cells were selected as the LF model in vitro; GA effectively inhibited the proliferation of LX-2 cells and induced apoptosis in a dose-dependent manner, and the mRNA and protein expression levels of α-SMA and collagen I were significantly downregulated. Further studies showed that GA significantly reversed LF and induced apoptosis in HSCs by blocking the PI3K/Akt, Bax/Bcl-2 and Wnt pathways [45,91]. After 12 weeks of GA treatment by gavage, the levels of HA, adhesion protein, serum total protein, albumin, alanine aminotransferase and aspartate aminotransferase were significantly reduced in a CCl4-induced rat model, which indicated that the reduction in oxidative stress levels could improve the state of LF. A study on the pathological mechanism showed that GA could significantly reduce the levels of malondialdehyde (MDA) and hydroxyproline and increase the activities of SOD and catalase in hepatic tissue [46]. GA can improve LF by scavenging free radicals, reducing lipid peroxidation, and inhibiting the activation and proliferation of HSCs. However, oral GA administration is associated with low bioavailability due to its water solubility and hydrophobicity, which limits its clinical use. Retinoic acid-modified acrylic nanoparticles were used to encapsulate GA, which significantly controlled its release and HSC targeting to improve the antifibrotic effect of GA on the liver [92].

#### 3.1.3. Silymarin

Silymarin (Figure 4C) is a polyphenol flavonoid antioxidant derived from plants that are mainly composed of flavonoid lignans, flavonoids and polyphenol molecules, and silybin is the most common and bioactive [93,94]. Related research shows that silymarin can protect the liver by reducing free radicals and lipid peroxidation [95]. After 10 days of 100 mg/kg silymarin treatment by gavage in a rat model induced by CCl4, MDA levels decreased and glutathione levels increased, indicating that silymarin has a significant antioxidant capacity and can protect the liver from damage. Studies have shown that silymarin can protect against NASH induced by an MCD diet by interfering with the inflammatory cytokine TNF-α, inhibiting the activation of HSCs, and reducing the expression of α1-procollagen in HSCs [47]. In addition, silymarin ameliorated LF by reducing the level of connective tissue growth factor in rats [48]. In an in vitro model of human LF, silybin dose-dependently inhibited the production of procollagen induced by growth factors in activated HSCs, resulting in antifibrotic effects [96]. The antifibrotic effect of silymarin has also been confirmed in humans. In a randomized, double-blind, placebo-controlled trial, compared with those in the placebo group, more patients in the silymarin 2100 mg/day group had measurable improvements in fibrosis. Noninvasive fibrosis indices (AST/platelet ratio index, fibrosis-4 score and NAFLD fibrosis score) in the silymarin group were significantly improved. In addition, there were more patients with fibrosis improvement or remission in the silymarin group, and silymarin changed liver stiffness favorably (the change in liver hardness was −0.7 vs. 6.0 kPa), but there was no significant difference between the two groups [97].

### 3.2. Polyphenol Compounds

Natural polyphenols are secondary metabolites of plants and have important roles in the prevention and treatment of many diseases, including cancer, cardiovascular disease, diabetes, aging and neurodegenerative diseases [98]. Studies have shown that polyphenols have a variety of pharmacological effects on oxidative stress, lipid metabolism, insulin resistance and inflammation, which are the most important pathological processes in the etiology of liver disease [98,99].

#### 3.2.1. Curcumin

**Figure 5:**
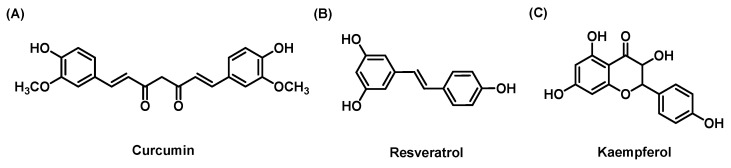
The chemical structures of (**A**) Curcumin; (**B**) Resveratrol; (**C**) Kaempferol.

Curcumin (Figure 5A) is a polyphenol compound isolated from *Curcuma longa* that contains many functional antioxidant groups, including β-diketone groups, carbon–carbon double bonds and phenyl rings. Due to its ability to eliminate lipid free radicals in cell membranes and convert them to phenoxyl free radicals, curcumin is considered to be a strong fat-soluble antioxidant [100]. It was found that curcumin (200 mg/kg/day for 3 weeks) protected against NASH induced by CCl4, and decreases in lipid accumulation and MDA deposition in histopathology were observed [49]. Curcumin also inhibited the occurrence and progression of LF in NASH mice induced by an MCD diet, which was characterized by a decrease in the secretion of TIMP-1 and the inhibition of 8-OH-deoxyguanosine-mediated liver oxidative stress in HSCs [50]. In addition, the protein expression of nuclear factor-erythroid 2-related factor 2 (Nrf2) in curcumin-treated rats increased significantly, suggesting that the prevention/improvement of NASH may be related to the activation of NRF2 [51]. An innovative mouse model of NASH and hepatocellular carcinoma (HCC) was used to study the potential mechanism by which curcumin can treat NASH. The results showed that curcumin improved hepatic steatosis and fibrosis in mice and caused a significant decrease in fibrosis biomarkers. The most important discovery was that curcumin inhibited the translocation of high mobility group protein B1 (HMGB1)-NF-κB, thus preventing NASH progression and hepatic injury [52].

Curcumin has been proven to have antifibrotic effects in various LF models, and its mechanism includes (1) inhibiting TGF-β/Smad signal transduction by activating autophagy, effectively reducing the occurrence of EMT in hepatocytes and inhibiting the production of ECM [101]; (2) reducing the phosphorylation of JNK and Smad3, inhibiting the activation of HSCs and inducing their apoptosis [102,103]; (3) decreasing the expression of HIF-1α through the ERK pathway [104]; (4) reversing LF by downregulating DNMT1, α-SMA and COL1A1 and demethylating key genes [105]; (5) inhibiting the activation of Kupffer cells (KCs) and reducing the secretion of chemokines to reduce the infiltration of monocytes [106]; and (6) targeting HSCs through a PPAR-γ activation-dependent mechanism to weaken sinus angiogenesis in LF [107].

#### 3.2.2. Resveratrol

Resveratrol (3,5,4′-trihydroxy-trans-stilbene, Figure 5B), which is a nonflavonoid phenol first isolated from *Veratrum grandiflorum*, has antiaging, anticancer, anti-inflammatory and antioxidant effects [108]. Resveratrol plays an interesting role in regulating the formation and deposition of new fibers. Resveratrol treatment by gavage reduced portal vein pressure and improved hepatic endothelial function in cirrhotic rats [109,110]. After resveratrol (10 mg/kg/day and 20 mg/kg/day) administration for 2 weeks, portal vein pressure decreased in cirrhotic rats, which was related to a decrease in thromboxane A2 and an increase in endothelial NO synthesis, which in turn was associated with a significant decrease in LF [53,54]. Resveratrol can prevent LF in various animal models, and the thickening and deposition of collagen fibers are significantly reduced in rats that are pretreated with resveratrol. Supplementation with resveratrol before dimethylnitrosamine (DMN) induction can significantly improve fibrosis, vasodilation, congestion, wall thickening, duct proliferation and necrosis [55]. The mechanism may involve decreasing the levels of MDA and the quantity of reduced glutathione (GSH), increasing the levels of GPx and SOD, and inhibiting the mRNA expression of inflammatory mediators, including inducible NO, TNF-α and IL-1β, and hypoxia-inducible factor-1α (HIF-1α) [111,112,113,114]. A recent study showed that resveratrol activated the PTEN/PI3K/AKT axis to alleviate LF in rats, and autophagy was enhanced after RSV treatment. In addition, resveratrol reversed the inhibitory effect of miR-20a on PTEN expression, decreased the expression of miR-20a, and promoted the protein expression of PTEN, PI3K and p-AKT, thus weakening LF [54].

#### 3.2.3. Kaempferol

Kaempferol (Figure 5C) is the most common glycoside flavonoid widely distributed in foods, beverages and the plant kingdom [115]. Kaempferol and its glycosylated derivatives have cardioprotective, neuroprotective, anti-inflammatory, antidiabetic, antioxidant, antitumor and anticancer effects [116,117]. Kaempferol has recently attracted much attention because of its multitarget characteristics and its potential for preventing and treating NAFLD [118]. In oleic acid-induced HepG2 cells and HFD-induced rats, kaempferol inhibited the NF-κB pathway and significantly reduced the levels of TNF-α and IL-6, thus significantly improving LF [119]. Some studies have shown that kaempferol significantly improves the number of inflammatory cells in the necrotic area of the hepatic lobule and central venules and reduces the levels of LN and HA. Protein analysis showed that kaempferol inhibited the development of LF by inhibiting the activation of HSCs. The Western blot results showed that kaempferol downregulated TGF-β1-induced α-SMA and the phosphorylation of Smad2/3 in a dose-dependent manner. In addition, kaempferol selectively binds to ALK5 and further downregulates the TGF-β1/Smad pathway [56]. Xing Wan et al. conducted similar research and reached a similar conclusion, but the new discovery here was that kaempferol reduced liver inflammation and fibrosis by inhibiting the TNF-α/NF-κB pathway [57].

### 3.3. Terpenoids

There is increasing evidence that terpenoids can effectively inhibit the progression of NAFLD and play a therapeutic role in different stages of the disease, including improving lipid metabolism, inhibiting oxidative stress, inhibiting inflammation and preventing fibrosis [120].

#### 3.3.1. Geraniol

**Figure 6:**
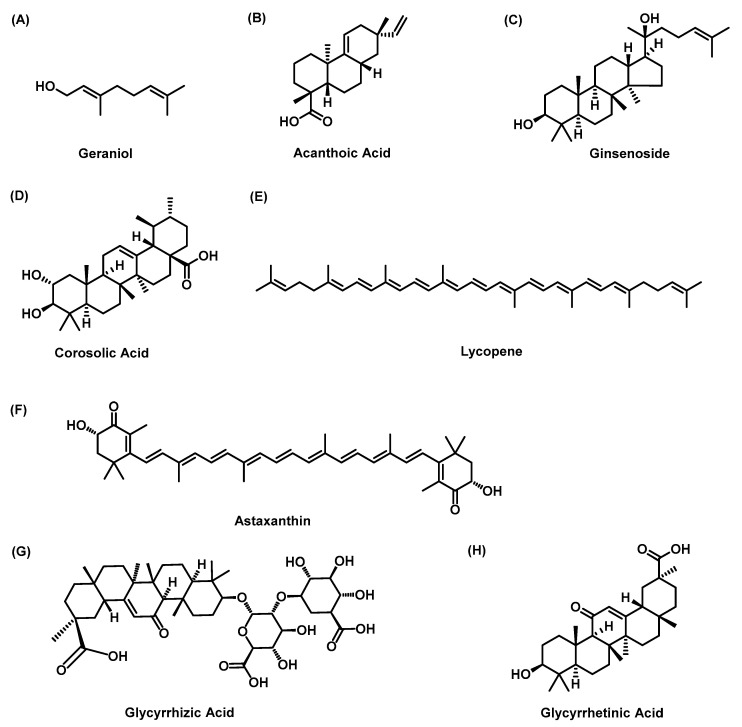
The chemical structures of (**A**) Geraniol; (**B**) Acanthoic Acid; (**C**) Ginsenoside; (**D**) Corosolic Acid; (**E**) Lycopene; (**F**) Astaxanthin; (**G**) Glycyrrhizic Acid; (**H**) Glycyrrhetinic Acid.

Geraniol (Figure 6A) is an acyclic isoprene monoterpene isolated from the essential oils of aromatic plants. In recent years, increasing evidence has shown that geraniol has an important antioxidant effect [121]. It has been reported that geraniol was effective in lowering the risk of hyperlipidemia in atherogenic diet-fed hamsters by improving endothelial function and preventing LF [122,123]. It was found that geraniol reduced the activity of myeloperoxidase and the protein expression of TNF-α and IL-6 in the livers of MCD-fed rats and significantly reduced the levels of COL1A1 and α-SMA [58]. In addition, geraniol increased the activities of GSH, SOD, catalase, glutathione reductase, glutathione-S-transferase (GST) and GSH-Px in the livers of rats and exerted antioxidant and anti-inflammatory effects [59].

#### 3.3.2. Acanthoic Acid

Acantholic acid (AA, Figure 6B) is a diterpene isolated from the root of *Eleutherococcus senticosus*. The treatment of liver diseases is an important aspect of the use of AA. The value of AA in liver diseases has been widely explored. For example, AA regulated LF and lipid deposition in HSC-T4 cells stimulated by ethanol combined with LPS by reducing lipoprotein2/4 through the TLR6 and IRAK1 signaling pathways [60]. AA also increased antioxidant enzymes and significantly reduced histopathological changes and the expression of caspase-3 and HIF-1α [61]. AA may be an attractive candidate for the treatment of NAFLD. Studies have shown that AA activates the farnesoid X receptor (FXR) and liver X receptor (LXR) signaling pathways and promotes the expression of the AMPK-SIRT1 signaling pathway, which plays a role in regulating fat metabolism and improving fibrosis [62].

#### 3.3.3. Ginsenoside

Ginsenosides (Figure 6C) are a series of glycosylated triterpenoids isolated and identified from the dry root and rhizome of Panax ginseng. Ginsenosides Rb1, Rb2, Rg1, Rg2, Rh1 and Mc1 have been proven to have protective effects on the liver [124]. Ginsenoside Rg1 is a phytochemical with biological activity, and it is the most commonly reported ginsenoside in the treatment of NAFLD [125]. Hou et al. showed that ginsenoside Rb1 alleviated LF by inhibiting fat deposition and the secretion of prostaglandin E2 and TIMP-1 [126]. Han et al. proposed that ginsenoside 25-OCH3-PPD could protect against LF and inflammation by activating the LXR signaling pathway in thioacetamide-induced mice. Compound K (CK) and ginsenoside Rh1 are the main metabolites of Panax notoginseng saponins (PNS) [63]. Previous studies have shown that PNS inhibits the activation of HSCs and LF by downregulating the expression of TIMP-1, collagen (PC)-I, PC-III and TGF-β1 [64]. A recent study showed that CK or Rh1 alone or in combination significantly improved liver damage caused by an HFD. Histologically, CK and Rh1 significantly reversed hepatocyte injury and LF induced by the HFD. In vitro, CK or Rh1 alone or in combination significantly induced apoptosis in HSC-T6 cells and inhibited cell proliferation and activation. In addition, CK and Rh1 alone or in combination inhibited the expression of TIMP-1, PC-I and PC-III. These results showed that CK and Rh1 had positive effects on NAFLD through antifibrotic and hepatoprotective activities [65].

#### 3.3.4. Corosolic Acid

Corosolic acid (CA, Figure 6D), a natural pentacyclic triterpenoid extracted from *Lagerstroemia speciosa* L. leaf, has efficacy in producing antidiabetic, anti-obesity, anti-inflammatory, antihyperlipidemic and antiviral effects [127,128]. In mouse models of NASH induced by HFD and CCl4, CA inhibits the transcription of profibrotic markers (including α-SMA, PC-1 and TIMP-1) and proinflammatory cytokines (including TNF-α, IL-1β, caspase-1 and IL-6) related to LF. CA also inhibits NF-κB translocation and the TGF-β1/Smad2 and AMPK pathways. In addition, CA decreased the expression of α-SMA and PC-1 and the phosphorylation level of Smad2 in LX2 cells treated with TGF-β1. The results showed that CA could improve fibrosis associated with NASH by regulating the TGF-β1/Smad2, NF-κB and AMPK signaling pathways [66].

#### 3.3.5. Lycopene

Lycopene (Figure 6E) is a lipophilic carotenoid hydrocarbon pigment found in red, pink, and orange fruit and vegetables [129]. Lycopene prevented the development of NASH induced by lipotoxicity by reducing oxidative stress in mice. Lycopene reduced the activity of peritoneal macrophages induced by LPS-/IFN-γ-/TNF-α and the expression of fibrotic genes in HSCs induced by TGF-β1 [67]. Lycopene has hepatoprotective and antioxidant effects in the context of NAFLD, and downregulating the expression of TNF-α and CYP2E1 may be one of the mechanisms [68]. Kitade et al. showed that lycopene improved LF by inhibiting the activity of HSCs [69]. In the same context, lycopene inhibited the activation of HSCs and regulated cell lipid storage by promoting the expression of PPAR-α and retinoid X receptor-β and -γ [130].

#### 3.3.6. Astaxanthin

Astaxanthin (ASTX, Figure 6F) is a kind of ketocarotene belonging to the tetraterpenes and has the strongest ability to absorb oxygen free radicals. Its antioxidant activity is higher than that of carotene, which is 1000 times that of vitamin E [131]. Natural astaxanthin is extracted from the green algae *Haematococcus pluvialis*, the red yeast *Phaffia rhodozyma* as well as crustacean byproducts [70]. Studies have shown that ASTX plays an important role in the prevention and treatment of LF, NAFLD, liver cancer and liver injury caused by drugs and ischemia and has therapeutic potential in both healthy and diseased livers [71]. ASTX inhibited the activation of the Smad3 pathway in HSCs by blocking the TGF-β1 signaling pathway [132]. In addition, ASTX decreased the activation of KCs and HSCs and increased the ratio of M1 macrophages to KCs in a mouse model of MCD-induced NASH. In addition, ASTX inhibited the expression of the fibrosis-related genes TGF-β1, Col1A1 and PAI-1 and alleviated liver inflammation and fibrosis [133]. These results indicate that ASTX may be a new and promising treatment for NASH.

#### 3.3.7. Glycyrrhizic Acid and Glycyrrhetinic Acid

Glycyrrhizic acid (GL, Figure 6G) and glycyrrhetinic acid (GA, Figure 6H) are the main bioactive compounds extracted from *Glycyrrhiza uralensis* Fisch and have been widely used for antitumor, anti-inflammatory, antiviral, and hepatoprotective purposes and for portal hypertension relief [72,73]. A mouse model of NASH induced by the MCD diet showed that glycyrrhizic acid and glycyrrhetinic acid inhibited deoxycholic acid-induced NLRP3 inflammasome-associated inflammation and blocked the mutual FXR-NLRP3 inflammasome pathways, significantly improving collagen deposition and decreasing the expression of α-SMA. Glycyrrhizic acid also significantly inhibited the mRNA expression of TGF-β1, TIMP1 and 2, collagen 1 and 2 and other fibrotic genes [74]. It was found that glycyrrhizic acid and its metabolite glycyrrhetinic acid inhibited the transcription of PC-I mediated by Smad3 and the activation of Q-HSCs in primary cultures and LF [75,134]. Glycyrrhizic acid also regulates the CD4^+^ T-cell response during liver fibrogenesis via the JNK, ERK and PI3K/AKT pathways [135].

## 4. Others

### 4.1. Calycosin

**Figure 7:**
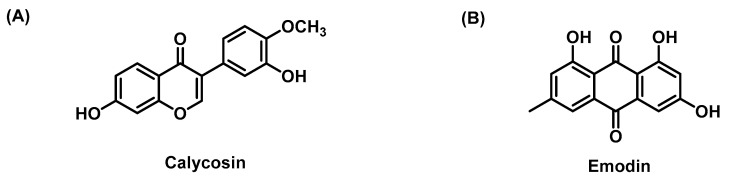
The chemical structures of (**A**) Calycosin; (**B**) Emodin.

Calycosin (Figure 7A) is a phytoestrogen with a similar structure to mammalian estrogen that is extracted from the root of *Astragalus membranaceus*. Pharmacological research and clinical practice have proven that calycosin improves triglyceride metabolism and antioxidant free radicals, inhibits liver injury, regulates glucose uptake disorders in hepatocytes, and inhibits HCC [76,77,136,137,138]. Overexpression of ERβ or calycosin alone inhibited the proliferation and migration of LX-2 cells induced by TGF-β1, downregulated α-SMA, PC-I, TIMP-1, and p-STAT3 and upregulated the protein expression of matrix metalloproteinase (MMP)-1. There was positive feedback between ERβ and calycosin. ERβ may inhibit the main functions of LX-3 cells by inhibiting the phosphorylation of STAT2, which is an important way for calycosin to inhibit liver fibrosis [78]. In addition, calycosin inhibited LF by balancing the MMP-1/TIMP-1 system, increasing the expression of Erβ and activating the JAK2-STAT3 pathway [139]. In addition, a rat model of MCD-induced NASH showed that calycosin inhibited the activation of HSCs by activating FXR and promoted the expression of PPARa, CPT1, Syndecan-1 and LPL, which are involved in the β-oxidation of free fatty acids, thereby reducing triglyceride accumulation and LF [140].

### 4.2. Emodin

Emodin (Figure 7B) is a compound extracted from rhubarb. Emodin is widely used to treat cardiovascular diseases, asthma, cancer, diabetes and organ fibrosis [79,80,141]. In an MCD-induced mouse model, emodin improved hepatic function, serum inflammation, histopathological inflammation scores and LF by inhibiting the expression of NLRP3 and the assembly of NLRP3 inflammatory bodies [81]. It was found that emodin inhibited the activation of HSCs by inhibiting the mRNA expression of TGF-β1, Smad4 and α-SMA [82]. In addition, emodin induced HSC apoptosis through the p53/ERK/p38 axis [142]. Oxidative stress is one of the pathological factors of LF. YAP1 is the main downstream target mediating oxidative stress. Emodin inhibited the phosphorylation of YAP1 and the generation of oxidative stress by reducing the expression of YES1 and AMPK, thus alleviating liver injury and slowing the occurrence of LF [143].

## 5. Conclusions and Perspectives

LF, which is the prepathological state of various liver diseases, such as cirrhosis and HCC, has become the key to effectively preventing and treating liver diseases. Natural products have stable curative effects and high safety and tolerance. Therefore, natural products with the ability to improve LF are gradually being discovered and studied. In this paper, natural products that inhibit hepatic fibrosis were summarized, and their mechanisms were analyzed in detail. The results showed that the natural products inhibited hepatic fibrosis mainly by blocking the Hedgehog, Wnt/β-catenin, TGF-β1 and NF-κB signaling pathways, enhancing the activities of SOD and GSH-Px, inhibiting the activities of TGF-β1, IL-1β, PPAR-γ and TNF-α, and decreasing the levels of MDA and TIMP1. At present, research on natural products to improve LF is mainly based on animal models. Therefore, their clinical application value should be explored in follow-up research to provide a reference for the clinical use of natural products to treat LF.

## Figures and Tables

**Table 1:** Pharmacological effects of natural products with anti-fibrotic activity in NAFLD.

Natural Products	Resource	Interfering Mechanism	Model	Pharmacological Effects	Refs
Baicalin	Flavonoids	NF-κB, Wnt, PPAR-γ	Mice	Antioxidant, anti-inflammatory and hepatoprotective	[42,43,44]
Galangin	Flavonoids	PI3K/Akt, Bax/Bcl-2, Wnt	LX-2 cell	Scavenges free radicals, reduces lipid peroxidation, inhibits the activation and proliferation of HSCs	[45,46]
Silymarin	Flavonoids	TNF-α, connective tissue growth factor	Rats	Reduces free radicals and lipid peroxidation	[47,48]
Curcumin	Polyphenol compounds	TGF-β/Smad, JNK/Smad3, ERK, PPAR-γ	Mice	Antioxidant and antifibrotic	[49,50,51,52]
Resveratrol	Polyphenol compounds	GPx/SOD, PTEN/PI3K/AKT	Rats	Anti-inflammatory and antioxidant	[53,54,55]
Kaempferol	Polyphenol compounds	TGF-β1/Smad2/3, TNF-α/NF-κB	Rats	Anti-inflammatory, antioxidant,	[56,57]
Geraniol	Terpenoids	TNF-α, IL-6, GPx/SOD	Rats	Antioxidant and anti-inflammatory	[58,59]
Acanthoic Acid	Terpenoids	FXR/LXR-AMPK-SIRT1	Mice and HSC-T6 cells	Antifibrotic	[60,61,62]
Ginsenoside	Terpenoids	LXR, TGF-β1	Mice and HSC-T6 cells	Anti-inflammatory and antifibrotic	[63,64,65]
Corosolic Acid	Terpenoids	NF-κ, TGF-β1/Smad2, AMPK	Mice	Anti-obesity, anti-inflammatory, antihyperlipidemic	[66]
Lycopene	Terpenoids	TNF-α, PPAR-α and RXR-β/γ	Rats	Antioxidant and antifibrotic	[67,68,69]
Astaxanthin	Terpenoids	TGF-β1/Smad3,	Mice	Antioxidant	[70,71]
Glycyrrhizic Acid	Terpenoids	FXR-NLRP3, JNK, ERK, PI3K/AKT	Rats	Anti-inflammatory	[72,73,74,75]
Glycyrrhetinic Acid	Terpenoids	FXR-NLRP3	Rats	Anti-inflammatory, hepatoprotective	[72]
Calycosin	Isoflavone	TGF-β1, Erβ, JAK2-STAT3, FXR	LX-2 cells, rats	Improves triglyceride metabolism and antioxidant free radicals, inhibits liver injury	[76,77,78]
Emodin	Isoflavone	TGF-β1, p53/ERK/p38, YAP1	Mice	Antioxidant and antifibrotic	[79,80,81,82]

## Data Availability

Not applicable.
